# **Impacts of** ***H. pylori*** **mixed-infection and heteroresistance on clinical outcomes**

**Published:** 2015

**Authors:** Masoud Alebouyeh, Abbas Yadegar, Nastaran Farzi, Marzyieh Miri, Homayoon Zojaji, Somayeh Gharibi, Zeinab Fazeli, Naser Ebrahimi Daryani, Hamid Asadzadeh Aghdaei, Mohammad Reza Zali

**Affiliations:** 1 *Foodborne and Waterborne Diseases Research Center, Research Institute for Gastroenterology and Liver Diseases, Shahid Beheshti University of Medical Sciences, Tehran, Iran*; 2 *Gastroenterology and Liver Diseases Research Center, Research Institute for Gastroenterology and Liver Diseases, Shahid Beheshti University of Medical Sciences, Tehran, Iran*; 3 *Basic and Molecular Epidemiology of Gastrointestinal Disorders Research Center, Research Institute for Gastroenterology and Liver Diseases, Shahid Beheshti University of Medical Sciences, Tehran, Iran*; 4 *Department of Internal Medicine, Division of Gastroenterology, Imam Khomeini Hospital, Tehran University of Medical Sciences, Tehran, Iran*

*Helicobacter pylori *infection is recognized as a major factor in the pathogenesis of chronic gastritis, peptic ulcer and gastric cancer (1, 2). Although treatment regimens containing a proton pump inhibitor (PPI) and combination of two or more antibiotics (amoxicillin, clarithromycin, metronidazole, or tetracycline) are considered to be the most efficacious, current antibiotic regimens show only 60–85% cure rates in clinical practices (3). The treatment failure was due to a pre-existing antibiotic resistant *H. pylori *strain or emergence of a new resistant strain from a susceptible ancestor. *H. pylori *resistance to amoxicillin, clarithromycin, metronidazole and tetracycline has been reported from Iran and other countries (4-6). Although *H. pylori *strains from individual patients typically have either an antibiotic susceptible or resistant phenotype, both antibiotic susceptible and resistant strains could exist among a single population of *H. pylori* in each patient. Heteroresistance represents infection with variants of *H. pylori *strains that were evolved during long-time chronic infection. Heteroresistance is believed to be as a cause of treatment failure of conventional therapeutic regimens against this infection (7-10). In a study in Iran, high frequency of mixed-type infection (77%) and also quasispecies development (15.4%) was reported, which suggests inefficacy of common therapeutic regimens in the infected patients due to co-existence of the resistant strains (11). Susceptible or heteroresistance strains could simultaneously present in the same region of the stomach (7), while their presence in different regions (antrum compared to corpus) was also reported (12, 13).

Failure of conventional therapy facilitates the emergence of strains with multidrug resistance (MDR) phenotypes. Several studies reported different rates of MDR phenotypes in patients with chronic infection. Nearly, 8.9-34.5%, 0.6-10.5%, and 15% of the strains in Asia, Europe, and America represent MDR phenotype (6, 14-16). Although regarding some of the regimens, such as rifabutin-based high-dose proton-pump inhibitor and amoxicillin triple regimen, which were reported to be effective against multidrug-resistant *H. pylori* strains, *H. pylori* eradication in patients who are infected with failed first and second eradication therapies seems to be difficult (17). The accuracy of information on *H. pylori *antimicrobial resistance is crucial to guide the selection of primary as well as secondary backup treatment regimens. Antimicrobial resistance is also important in the selection of an appropriate regimen to avoid development of higher level of resistance to antibiotics that makes the retreatment difficult.

Emergence of resistant strains of *H. pylori* from the susceptible parental strain could be concomitant with the change in virulence property of the bacterium in the gastric tissue (11). Changes of *H. pylori* virulence genotype could happen under stress conditions, e.g. antibiotic selective pressure, which promotes nucleotide substitution, insertion, deletion and recombination in *H. pylori *genomic DNA (18). This event is worrying, because the infected patients will be confronted with the most virulent strains that are resistant to conventional treatment regimens.

Except heteroresistance and mixed infection, other factors are also involved in the occurrence of* H. pylori* eradication failure, including a high count of the colonized bacteria, the gastric pH (gastric acid hyper-secretion), smoking, non-ulcer diseases, gastroduodenal diseases, pangastritis, *H. pylori *intracellular life, colonization in niches with low antibiotic penetration, dormancy or low metabolism of the strains during the treatment phase, re-infection, virulence property, recombination rate, obesity, and polymorphisms of CYP450 (19). Designation of effective regimens to remediate all the factors that affect treatment success in the infected patients seems to be hard.

The heteroresistance for different antibiotics varied from the lowest one that usually was seen for amoxicillin to the highest one that was seen for metronidazole (8). This phenotype was also reported for tetracycline and clarithromycin. Heteroresistance of *H. pylori* isolates by minor genomic alterations from a pre-existing *H. pylori* strain with a single genotype needs special attention, and this is especially challenging for the discovery of new therapeutic approaches. This could happen with instability of the genome during long-term evolution. Reinfection with *H. pylori* following successful bacterial cure is unusual in developed countries (20). It was estimated that, reacquisition of the bacterium occurs in <2%/persons/year, which is similar to the rate of primary infection among adults in these countries (21, 22). This rate is higher in developing countries (13.0% versus 2.67%, respectively) (23, 24).

Failure of *H. pylori* eradication is associated with diverse clinical outcomes, including chronic gastritis, hypergastrinemia, ulcer formation, intestinal metaplasia, atrophy, dystrophy, obesity, insulin resistance and cancer in the infected patients (25-32). The prolonged inflammatory reaction is believed to be one of the main factors contributing to the malignant transformation of the gastric epithelium in these patients. *H. pylori* eradication prevents development of these preneoplastic changes of the gastric mucosa, including atrophic gastritis and intestinal metaplasia (33-35). A randomized control study by Zhou et al. showed regression of gastric precancerous lesions by *H. pylori* eradication (36). In patients with peptic ulcer disease, eradication failure is associated with a 60% annual ulcer recurrence rate and increased risk of gastric adenocarcinoma compared with 10% after successful eradication (37). Eradication of *H. pylori* infection was recommended in patients with gastritis. There are a number of studies showing the benefit of anti-*H. pylori* treatment in reducing both the progression of precancerous lesions and the risk of gastric cancer (38).

**Which therapeutic strategy should be used against the heterorsistant** ***H. pylori*** **infection?**

Although recent improvements have been obtained in the efficacy of treatment regimens against *H. pylori* infection, nearly all designs present some margin of failure in eradicating the bacteria (39). Therefore, a treatment strategy should be specifically considered for eradication of these bacteria. Primary resistance of *H. pylori* strains towards antibiotics prescribed in the current eradication regimens affects the therapeutic outcome. Decreased cure rate of *H. pylori* infection in patients with eradication failure due to infection with heteroresistant strains compared with pure susceptible strains was reported in one study (40). It has been shown that the presence of either clarithromycin or metronidazole resistance considerably reduces the success rate of first-line anti-*H. pylori* therapy (41). Based on these observations, European guidelines suggest to prolong amoxicillin-based standard triple therapy to 14 days where primary clarithromycin resistance is >15-20%, if primary metronidazole resistance is >40% (42). According to the 4^th^ edition of the Maastricht consensus report, in regions with high clarithromycin resistance rate (>20%), first line therapy should be performed by bismuth quadruple therapy, or non-bismuth containing regimes (either concomitant or sequential therapy). PPI-levofloxacin-amoxcicillin based regimens were also recommended for the second line therapy. However, it was reported that third-line treatment should be chosen according to the local resistance patterns based on the culture and antibiotic susceptibility testing results (43).

In conclusion, it is important for physicians to be informed about the rate of resistance to commonly prescribed antibiotics against *H. pylori* in their regions. Mixed infection due to heteroresistant strains or co-infection with distinct strains should be considered in patients who experienced chronic *H. pylori* infection with a history of eradication failure. Assessment of the failure rate of treatment regimens in geographic areas with higher frequency of resistant strains seems to be necessary. Since different antibiotic resistance patterns are seen in isolated strains from different gastric sites, sampling of biopsy specimens should be performed from both antrum and corpus regions for antimicrobial susceptibility testing. Appropriate breakpoints should be chosen in each country for interpretation of the antibiotic susceptibility results for the selection of optimal regimens in these patients.
